# Maintained physical activity and physiotherapy in the management of distal upper limb pain – a protocol for a randomised controlled trial (the arm pain trial)

**DOI:** 10.1186/1471-2474-15-71

**Published:** 2014-03-10

**Authors:** Gareth T Jones, Kathrin Mertens, Gary J Macfarlane, Keith T Palmer, David Coggon, Karen Walker-Bone, Kim Burton, Peter J Heine, Candy McCabe, Paul McNamee, Alex McConnachie

**Affiliations:** 1 Musculoskeletal Research Collaboration (Epidemiology Group), University of Aberdeen, Aberdeen, UK; 2 MRC Lifecourse Epidemiology Unit, University of Southampton, Southampton, UK; 3 Centre for Health and Social Care Research, University of Huddersfield, Huddersfield, UK; 4 Warwick Clinical Trials Unit, University of Warwick, Coventry, UK; 5 The Royal National Hospital for Rheumatic Diseases NHS Foundation Trust, Bath, UK; 6 University of West of England, Bristol, UK; 7 Health Economics Research Unit, University of Aberdeen, Aberdeen, UK; 8 Robertson Centre for Biostatistics, University of Glasgow, Glasgow, UK; 9 Epidemiology Group, Institute of Applied Health Sciences, University of Aberdeen, School of Medicine and Dentistry, Polwarth Building, Foresterhill, Aberdeen AB25 2ZD, UK

**Keywords:** Randomised controlled trial, Arm pain, Physiotherapy, Advice, Rest, Active, Pain management

## Abstract

**Background:**

Distal upper limb pain (pain affecting the elbow, forearm, wrist, or hand) can be non-specific, or can arise from specific musculoskeletal disorders. It is clinically important and costly, the best approach to clinical management is unclear. Physiotherapy is the standard treatment and, while awaiting treatment, advice is often given to rest and avoid strenuous activities, but there is no evidence base to support these strategies. This paper describes the protocol of a randomised controlled trial to determine, among patients awaiting physiotherapy for distal arm pain, (a) whether advice to remain active and maintain usual activities results in a long-term reduction in arm pain and disability, compared with advice to rest; and (b) whether immediate physiotherapy results in a long-term reduction in arm pain and disability, compared with physiotherapy delivered after a seven week waiting list period.

**Methods/Design:**

Between January 2012 and January 2014, new referrals to 14 out-patient physiotherapy departments were screened for potential eligibility. Eligible and consenting patients were randomly allocated to one of the following three groups in equal numbers: 1) advice to remain active, 2) advice to rest, 3) immediate physiotherapy. Patients were and followed up at 6, 13, and 26 weeks post-randomisation by self-complete postal questionnaire and, at six weeks, patients who had not received physiotherapy were offered it at this time. The primary outcome is the proportion of patients free of disability at 26 weeks, as determined by the modified DASH (Disabilities of the Arm, Shoulder and Hand) questionnaire.

We hypothesise (a) that advice to maintain usual activities while awaiting physiotherapy will be superior than advice to rest the arm; and (b) that fast-track physiotherapy will be superior to normal (waiting list) physiotherapy. These hypotheses will be examined using an intention-to-treat analysis.

**Discussion:**

Results from this trial will contribute to the evidence base underpinning the clinical management of patients with distal upper limb pain, and in particular, will provide guidance on whether they should be advised to rest the arm or remain active within the limits imposed by their symptoms.

**Trial registration:**

Registered on http://www.controlled-trials.com (reference number: ISRCTN79085082).

## Background

Upper limb pain is common and responsible for considerable disability, demand for health care and lost productivity. Among working-aged respondents to a recent population survey in England, 15% had consulted a GP within the past year with upper limb pain [1]; 5% had seen a specialist; 14% reported symptoms persisting more than six months; and 10% reported disabling pain [2]. Further, data from the UK Labour Force Survey indicate that work-attributed cases of upper limb disorder cause an estimated annual loss of 4.7 million working days [3]. Upper limb pain is more likely to become persistent if accompanied by poor mental health, adverse psychosocial factors, including a somatising tendency (tendency to be distressed by common physical symptoms), and adverse beliefs concerning health and activity [4,5]. Indeed, it is plausible that response to treatment may vary importantly between subgroups defined by such characteristics.

Distal upper limb pain is defined as pain in the elbow, forearm, wrist or hand, may be non-specific in origin, or may arise from a number of specific disorders (e.g. medial and lateral epicondylitis, and tenosynovitis). In a study of patients presenting to primary care and physiotherapy services with upper limb pain, 42% of patients had pain in the distal arm and at twelve months 48% were still in pain, and 19% reported unremitting pain (never pain-free for as long as seven consecutive days) [5]. Although distal upper limb pain is clinically important, and costly, the best approach to managing symptoms is unclear.

### Evidence for the effectiveness of advice in the treatment of upper limb pain

Patients with upper limb pain are commonly referred to physiotherapy and, while awaiting treatment, are often advised to rest the arm and avoid purported harmful activities as a precautionary measure. Indeed, online health advice offered by the UK National Health Service (NHS) states that ‘it is likely that your GP will probably advise you to temporarily stop doing the task or activity that is causing your symptoms’ [6]. Similarly, many employers and occupational physicians are wary of allowing employees with distal upper limb pain to continue with forceful or repetitive work activities for fear of possible litigation. However, such advice lacks an evidence base. A search of MEDLINE and BIDS Embase in 2009 (at the time the funding application for the current trial was prepared) confirmed that no randomised controlled trial has hitherto investigated the benefits of resting the painful arm as compared with remaining active within the limits of pain. This omission is both important and surprising. An analogy can be drawn with non-specific low back pain, which shares many aetiological and prognostic factors and which for many years was managed by bed rest. However, trials – and, latterly, large-scale community health campaigns – have demonstrated that back pain prognosis is improved if patients are advised to remain active [7,8].

Written information providing evidence-based advice to patients with back pain (*The Back Book*) has been shown to be effective in promoting positive beliefs and contributing to improved clinical outcomes [9,10]. The way in which the advice is imparted is based firmly on a biopsychosocial approach: traditional beliefs about the relationship between activity and pain are challenged, the benefits of remaining active are contrasted with the disadvantages of undue rest, and individuals are empowered to take responsibility for their recovery. This differs conceptually from a biomedical approach in which the patient is a passive recipient who is told what has gone wrong (injury), how it is diagnosed, and what healthcare can do to fix it. The activity-promotion approach has been extended to the management of neck injury (*The Whiplash Book*), where a booklet focusing on reducing anxiety about the injury, and stressing the benefits of remaining active, has been well received by patients and caused a positive shift in beliefs [11]. More recently available is *The Arm Book* which also provides evidence based advice on how to deal with upper limb pain or injury. However, its effectiveness is currently unproven [12].

It seems plausible that patients with distal upper limb pain might benefit from a similar approach to back pain. Many such cases (like cases of back pain and whiplash injury) arise in the absence of well-defined local pathology, and the natural history and predictors of arm pain share much in common with these other musculoskeletal disorders – fear-avoidance, health anxiety, pessimistic attitudes, psychological distress and adverse psychosocial factors [4,5,13]. We hypothesise a number of possible mechanisms through which we may hasten improvement in function, by challenging disadvantageous beliefs about ‘injury', reducing fear and distress, and encouraging self-management and the maintenance of an active approach to recovery. Whether patients with distal upper limb pain benefit from keeping active, as back pain sufferers do, is logically the next research question to address and well-conducted trials are needed to resolve these issues.

### Hypothesis/aims

The primary aim of this study is to investigate whether, among patients awaiting physiotherapy for distal upper limb pain (pain in the elbow, forearm, wrist or hand), advice to remain active and maintain usual activities results in a long-term reduction in upper limb pain and disability, compared with advice to rest.

Potential participants will be identified from patients with distal upper limb pain referred to physiotherapy. However, whether physiotherapy itself is efficacious in the management of distal upper limb pain is not well established. Therefore, in the context of the proposed trial, a pragmatic opportunity exists to determine the value of early physiotherapy in comparison with treatment that is delayed until the usual waiting list time. Therefore, a secondary aim is to determine, among the same patient population, whether immediate (‘fast-track’) physiotherapy results in a long-term reduction in upper limb pain and disability, compared with physiotherapy delivered at the usual waiting list time – typically, after a period of approximately seven weeks.

## Methods/design

### Study design overview

This paper presents the protocol for a multi-centre, randomised, controlled trial. Potentially eligible patients with distal upper limb pain are identified from waiting lists in out-patient physiotherapy departments. Recruited patients undergo telephone screening in the first instance, followed by a pre-trial assessment by a research nurse, to confirm eligibility and collect baseline information. Eligible patients are randomised to one of three groups:

• Group 1 – advice to remain active while awaiting physiotherapy treatment;

• Group 2 – advice to rest the arm while awaiting physiotherapy treatment; or

• Group 3 – immediate physiotherapy.

All participants are followed up by postal questionnaire 6, 13 and 26 weeks post-randomisation to determine arm pain and function. In addition, at six weeks, participants from Groups 1 and 2 who indicate that they still require physiotherapy are offered immediate treatment. Thus, this treatment will be delivered at around seven weeks equating, approximately, to that on a ‘usual care’ waiting list.

### Ethical issues

Ethical approval has been obtained from the South Central – Hampshire A Research Ethics Committee (11/SC/0107). All participants are required to provide written informed consent at initial assessment. Patients are required to consent to complete the study questionnaire, undergo the brief examination and, if eligible, to be randomised into one of the treatment groups. Participants are not being made aware that there are two different types of advice to prevent compromise of the advice being given.

### Identification of potential participants

From January 2012 to January 2014, male and female patients aged 18 years or older were identified from out-patient physiotherapy services in fourteen UK trial sites (see Table 1). Eligibility was established by screening referral letters for physiotherapy and confirmed by questioning and physical examination carried out by trained research nurses during an initial assessment visit. Patients were potentially eligible if they were referred (or self-referred) to out-patient physiotherapy for treatment of distal upper limb pain/disability. However, patients were excluded if they met one, or more, of the following criteria: (a) aged <18 yrs at the time of screening; (b) the patient had received previous physiotherapy for distal upper limb pain within the past twelve months; (c) the disorder was of a type for which physiotherapy of the distal upper limb was not the primary treatment (e.g. pain referred from the neck/shoulder); (d) the pain was due to a fracture, systemic inflammatory disease, or cancer; (e) the patient has complex regional pain syndrome; (f) symptoms were due to a specific condition for which advice to remain active is contraindicated (e.g. florid tenosynovitis); (g) the appointment was classed as an emergency; and/or (h) the patient was embroiled in a legal dispute regarding their arm pain.

**Table 1:** Trial recruitment sites

**Centre**	**First patient randomised**	**Last patient randomised**
Aberdeen	03-Feb-12	04-Feb-14
Southampton	20-Feb-12	29-Apr-13
Brighton	16-Mar-12	07-Feb-14
Sussex	17-Aug-12	27-Dec-13
Stockport	13-Sep-12	23-Jan-14
Newcastle	27-Nov-12	14-Jan-14
Southend	03-Dec-12	19-Nov-13
King’s college	01-Feb-13	28-Jan-14
Wigan	11-Feb-13	10-Feb-14
Huddersfield	14-Feb-13	23-Jan-14
Leicester	14-Mar-13	28-Nov-13
Birmingham	28-Mar-13	06-Jan-14
Bath	08-May-13	18-Feb-14
St Helens	16-May-13	08-Oct-13

### Initial assessment visit

Patients who attended the initial assessment were asked to self-complete a questionnaire which asked about demographic characteristics (age, sex, social class); employment circumstances (including occupational activities); symptom history (e.g. unilateral or bilateral symptoms, duration, disability) and all prior treatments to date. In addition, data were collected on general health, physical and mental well-being, fatigue, other symptoms (headache/abdominal pain/chronic widespread pain), somatic distress, health beliefs (especially fear avoidance) and smoking status.

Patients also underwent a standardised upper limb examination: the Southampton Examination Schedule for Upper Limb Disorders which includes inspection and palpation of the upper limbs, and clinical provocation tests (including Finkelstein’s test, Phalen’s test and Tinel’s test). The Schedule is based on a national Delphi consensus, has been validated in hospital out-patient and community settings, and has been applied in previous large-scale epidemiological studies [14,15].

Both questionnaire and examination assessed factors were considered as important prognostic markers, potential modifiers of treatment response, baseline measure of outcomes, or were important for randomisation and minimisation.

### Randomisation process and allocation concealment

Randomisation was conducted by the Robertson Centre for Biostatistics, a registered clinical trials unit at the University of Glasgow.

Patients were allocated to one of the three treatment groups using a mixed randomisation and minimisation algorithm to maintain treatment balance with respect to treatment centre, laterality (dominant, non-dominant, bilateral), a broad categorisation of diagnosis (elbow disorder, or wrist/hand disorder), and baseline arm function, as assessed using a modified DASH score (the primary outcome measure, with scores grouped as 0–5, 6–8, or 9–11). For each new patient, the minimisation variables were entered into a web-based data collection system, created by the trials unit; the system then allocated the patient to one of the three groups. One third of patients were allocated completely at random, whilst two-thirds were allocated according to the minimisation algorithm. Randomisation to the three groups, or entry to the minimisation procedure, was determined according to a pre-specified allocation schedule generated using the method of randomised permuted blocks of nine participants. This schedule was known only to a restricted group of staff at the trials unit. Within the minimisation algorithm, in the event of a tie between treatment groups (i.e. when allocation to more than one group would provide an equally low level of imbalance), treatment was allocated at random between the tied treatment groups.

### Blinding

Because of the nature of the interventions, it was not possible to blind study participants. However, to prevent ‘contamination’ it is important that participants were unaware of the differing advice given to others. Therefore, patients were informed that if they participated, they would be randomised to immediate physiotherapy or advice, without giving explicit details about the nature of the advice (or even the fact that there were two advice groups).

### Treatment groups

• Group 1 – advice to remain active;

Participants randomised to receive advice to remain active were given a seven-page leaflet focusing on positively shifting beliefs about activity and arm pain, along with practical advice on staying active. The messages were reinforced verbally by a research nurse using a brief standardised script. The experimental leaflet was developed from the findings of a recent Health and Safety Executive Research Report of a comprehensive review of arm pain [16]: this research confirmed that biopsychosocial principles apply to the management of work-related upper limb disorders and a number of evidence-based messages were proposed. Key among these were that upper limb pain is common; early return to work is helpful; lasting damage is rare; recovery and return to full activities can be expected; some cases may need treatment but many settle with self-management; and that maintaining activity is probably helpful.

These messages are very similar to those that apply to back and upper limb pain and are amenable to presentation written form - similar to *The Back Book*, *The Whiplash Book*, and *The Arm Book*[9,10,12]. Cognitive behavioural principles underlie the patient-centred information and advice, which focuses on the benefits of remaining active. The text was prepared by Burton and Kendall, who led the Health and Safety Executive project and have experience of producing this sort of patient educational material, with input from other members of the Arm Trial research team. The leaflet also underwent further review by several colleagues, and by a focus group of end users, to ensure accuracy, general acceptability and clarity.

• Group 2 – advice to rest the arm; or

Participants randomised to receive advice to rest the arm received a leaflet similar in length, design and appearance to the booklet for Group 1. This leaflet was based on material available via NHS Direct: it covers a range of diagnoses, can be taken to reflect current clinical practice, and contrasts with the approach adopted for the experimental leaflet (Group 1). The style is solidly biomedical and the advice is about rest and avoidance (as well as treatment), rather than maintaining activity.

• Group 3 – immediate physiotherapy.

Participants randomised to receive immediate physiotherapy were fast-tracked to treatment which they received at the earliest opportunity. Additional physiotherapy staff were provided in all participating trial centres so initial physiotherapy assessment (independent of the trial screening examination) and first treatment would occur in a matter of days, rather than weeks. Whilst this trial is intended to be pragmatic in so far as it is reflective of usual physiotherapy practice, we undertook additional work to ensure the treatment programmes were compliant with both the Medical Research Council’s and the CONSORT organisation’s guidance on developing and reporting complex interventions. In the early phases of the study we documented and developed the intervention to ensure it represents best usual care. This involved a review of appropriate treatment guidelines and the literature to ascertain current best practice. It also included discussions with physiotherapists involved in the trial in order to establish current practice and, if any differences were encountered, to reconcile the findings of the literature with actual clinical practice. A broad set of guidelines was developed in accordance with best practice which gave therapists the flexibility to treat patients on an individual basis without being overly prescriptive.

Further, it is important that the interventions be documented so they can be reported and replicated, and the physiotherapists who deliver the intervention be involved in this process (to facilitate compliance with the treatment protocol). Treatments were recorded using a standardised pro forma and record, for example, treatment modality, number and timing of appointments attended, and time to discharge.

### Follow-up and trial outcomes

Follow-up is still ongoing. Participants are followed up at 6, 13, and 26 weeks post-randomisation by self-complete postal questionnaires, and non-responders receive a reminder questionnaire after two weeks. After a further two weeks, non-responders are followed up by telephone, and are asked brief questions on the primary outcome only, using a standardised pro forma. In addition, at six weeks patients randomised to receive advice (Groups 1 or 2) receive a letter offering them a physiotherapy appointment which, if accepted, is received at the earliest opportunity. This approach, requiring an active opt-in to treatment, is consistent with current practice for patients not involved in the trial. Also, the delay between randomisation and the offer of appointment is equivalent to what would be usual care physiotherapy in most UK centres. Participants electing to receive physiotherapy are offered immediate treatment and receive that therapy, as per usual protocol (and as per Group 3), at the discretion of the treating physiotherapist. Although the precise treatment modalities given may differ between fast-track and delayed physiotherapy groups, this reflects usual care, where treatment choice may be dependent on time elapsed since initial referral and activities undertaken in the interim.

The DASH (Disabilities of the Arm, Shoulder and Hand) questionnaire is an instrument designed to measure physical function and symptoms in patients with single or multiple musculoskeletal disorders of the upper limb. It has been shown to have good test-retest reliability and validity. Further, it has high internal consistency, correlates well with other measures of arm function, and discriminates between persons who are/are not able to undertake activities of daily living, or work without restriction [17]. However, the DASH instrument has a number of important limitations in the context of the current trial. Specifically, (a) there is a lack of good quality reference data for the DASH from the United Kingdom; (b) the DASH instrument focuses on the whole of the upper limb and contains no items that are specific to the distal arm; (c) none of its elements relate explicitly to activity limited by pain; (d) the timeframe of inquiry (past seven days) is likely to limit study power for certain outcomes (e.g. difficulty changing a light-bulb) and is less relevant clinically in a trial designed to assess sustained recovery; and (e) some items are relevant only to subsets of the study population (e.g. preparing meals, making beds), rather than providing a question set based on activities that everyone is likely to perform.

**Figure 1:**
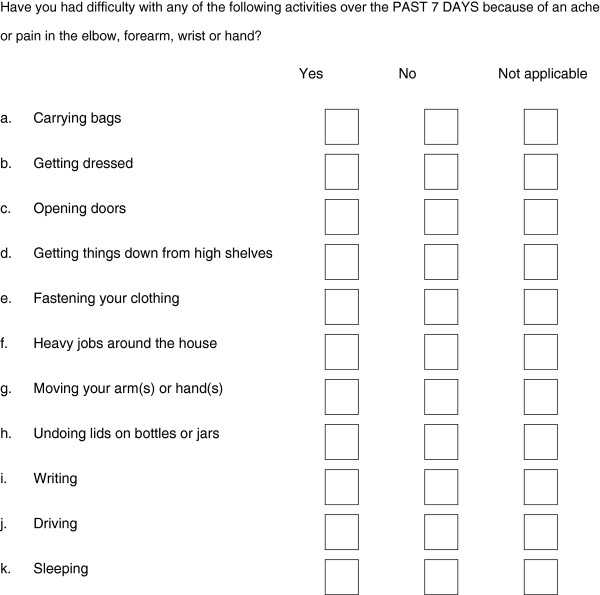
Modified DASH.

Accordingly, we developed a modified instrument to determine arm function. Based in part and in format on the original DASH questionnaire, and called here the 'modified DASH', this instrument asks participants to rate difficulty in performing eleven pre-specified activities over the previous seven days because of pain in their distal upper limb (Figure 1). This modified DASH, which has improved face validity over the DASH for our purposes, has previously been used in a large population survey examining the outcome and prognostic determinants for upper limb pain presenting in primary care and to physiotherapy services [5]. In this pilot phase, the instrument was shown to be sensitive to change, and to track clinical recovery over the time interval of interest. It enables a well-powered, clinically relevant dichotomous outcome (full recovery versus not) to be studied.

Thus, for the current trial, the primary outcome is the proportion of patients free of disability at 26 weeks post-randomisation, as determined by the modified DASH. As a secondary outcome for participants randomised to Group 1/Group 2, we aim to determine the proportion who still seek physiotherapy at six weeks. In addition, we collect information on the type of treatments given, and time to discharge. Other secondary outcomes, in all participants, include: upper limb pain and function, coping, fear of movement, ability to function at work, aspects of general health and quality of life.

In addition, health care costs will be collected from participants using structured questionnaires. These will record distal upper limb related hospital admissions, out-patient attendances, and visits to/from relevant health professionals. Published UK sources of data on unit cost will be applied to value use of resources. For EQ-5D and SF-12 outcomes, published UK tariffs will be used to convert these data to quality of life weights.

### Statistical issues

#### Proposed sample size/power calculation

The study is powered to determine a difference in the proportion of patients fully recovered at 26 weeks post-randomisation, as assessed using the modified DASH. The principal analysis will consist of a comparison between participants in Group 1 and Group 2 (advice to maintain usual activities, versus advice to rest the arm). We have previously shown that, among persons with distal arm pain undergoing usual care, 51% were free of disability at six months, with little further improvement (up to 60%) at 12 months [5]. The trial is powered on the assumption that 70% of participants in Group 1 will be free of disability at six months. We require 148 subjects in each group in order to detect this difference with 90% power and an alpha of 5%. From previous trials with similar follow-up methods we anticipate, conservatively, that 80% of patients randomised will complete a follow-up questionnaire at 26 weeks [18]. Thus, we need to randomise 185 participants per group. We intend, in addition, to recruit 185 participants into Group 3 (immediate physiotherapy). Thus, we aimed to randomise 555 participants in total.

#### Statistical analysis

Analysis will determine whether, at 26 weeks post-randomisation, patients in Group 1 (advice to remain active and maintain usual participation) experience an improvement in upper limb pain and function, compared with those in Group 2 (advice to rest the painful arm). In addition, we will examine whether, at 26 weeks post-randomisation, patients who received immediate physiotherapy (Group 3) experience an improvement in upper limb pain and function compared with those who received delayed treatment (from Groups 1 and 2 combined).

Estimation of the treatment effects will be based on intention-to-treat and will be conducted at the end of the follow-up period (26 weeks after the final randomisation). To address the primary research question a logistic regression model will be fitted to estimate the odds ratio for full recovery at 26 weeks post-randomisation between Group 1 and Group 2. The model will include treatment group (as a three-level categorical variable), age (as a continuous variable, or categorical, depending on the nature of any association), and as categorical variables: sex, study centre, pain location (elbow, wrist/hand, or both), laterality (dominant, non-dominant, or bilateral) and baseline modified DASH score (0–5, 6–8, or 9–11). If small numbers in any of these sub-groups causes problems for model convergence, alternative specifications will be considered.

For the primary analysis of the impact of advice to remain active compared to advice to rest, the method of recycled predictions (using 1000 bootstrap samples) will be used to estimate the absolute difference in the probability of being fully recovered between the two groups, with a 95% confidence interval.

Likewise, for the secondary research question, the impact of early compared to delayed physiotherapy, the method of recycled predictions (using 1000 bootstrap samples) will be used to estimate the absolute difference in the probability of being fully recovered between the early physiotherapy group and the two delayed physiotherapy groups combined, with a 95% confidence interval.

Although the main analysis will classify the modified DASH score dichotomously – i.e. fully recovered (zero disabilities) or not – as a secondary analysis the modified DASH score will be considered as a continuous variable. A linear regression model will be fitted, including the treatment group, age, sex, study centre, pain location, laterality and baseline modified DASH score (as a continuous variable). Again, if small numbers in any of these sub-groups causes problems for model convergence, alternative specifications will be considered. To assess the impact of advice to remain active compared with advice to rest, and to compare early with usual care physiotherapy, appropriate contrasts will be applied to the coefficients of the above model to estimate the adjusted mean differences in modified DASH scores between Groups 1 and 2, and between Group 3 and the mean of Groups 1 and 2 combined, with 95% confidence intervals.

The above analysis will be repeated using the same outcome measures collected at different assessment time points (6 and 13 weeks post-randomisation). In addition, evidence of treatment effect heterogeneity will be assessed by including terms for interactions between treatment and each of the other variables in the regression models.

Finally, a cost-utility analysis will be performed to assess the health care and patient costs, and quality of life effects, associated with provision of each of the three treatments. A series of incremental cost per Quality Adjusted Life Year (QALY) ratios will be calculated. Sensitivity analysis will be employed to quantify the uncertainty surrounding the calculated ratios. In addition, non-parametric methods will be used for calculating the confidence intervals around cost per QALY ratios, using bootstrapped estimates of the mean cost and QALY differences. To summarise cost-effectiveness, cost effectiveness acceptability curves will be employed to show the probability that different interventions are cost effective for different values of willingness to pay per additional QALY.

## Discussion

We have outlined the rationale and design of a randomised controlled trial to investigate whether, amongst patients awaiting physiotherapy treatment for distal upper limb pain, advice to remain active results in a long-term reduction in arm pain and disability, compared with advice to rest. Further, the trial gives rise to a pragmatic opportunity to examine whether, among the same patient population, immediate (‘fast-track’) physiotherapy results in a long-term reduction in arm pain and disability, compared with physiotherapy delivered at the usual time – typically, after a waiting list period of 6–8 weeks.

Patients awaiting physiotherapy for distal arm pain are commonly advised to rest the affected limb and avoid strenuous activities to ‘prevent further injury’. However, there is currently no sound evidence to support this strategy. The current trial is novel, in that it tries to address this, although similar strategies have been applied in other areas and have led to enormous clinical benefit. In low back pain, for >20 yrs the benefits of remaining active – as opposed to bed-rest, widely advocated at the time – have been well known. The evidence supporting maintenance of activity among persons with low back pain is now well established. Indeed recent studies from Australia have demonstrated that widespread publicity campaigns, promoting this message, result in a reduction in the number of claims for back pain compensation, and medical payments for back pain in the general population [19]. However, despite the epidemiological evidence to suggest that many regional pain syndromes share common aetiological and prognostic factors [13], there has been little extrapolation of these principles to other pain conditions.

The results of this trial will have an immediate influence on the management of patients with distal arm pain. Also, depending on the results, it may inform future research studies to refine management. For example, we may find that there is a worthwhile benefit from remaining active and maintaining usual activities while awaiting physiotherapy treatment. If this is the case, this would provide strong evidence that the advice currently given to patients with distal arm pain should be changed. Alternatively, if there is no additional benefit from advice to remain active, this would support the current treatment strategies and current advice that is given. It would also provide the stimulus for examining in more detail the differences between low back pain and distal arm pain – i.e. why, when the risk factors for long-term pain and disability are similar, do similar treatment approaches fail to work?

In addition, we may find that ‘fast-track’ physiotherapy has beneficial outcomes, compared to physiotherapy delivered at the usual time. This would provide good evidence in support of a rapid-access policy which, in turn, would translate into clear and immediate patient benefit. It would be unethical to conduct a study where some patients received physiotherapy and others did not. However, if the current trial demonstrates that there is no additional benefit from early physiotherapy, this would provide the rationale (and the ethical justification) for randomised trials of specific physiotherapeutic modalities. This might lead to more refined physiotherapy treatments and more efficient use of healthcare resources.

### Ethics

South Central – Hampshire A Research Ethics Committee (reference number: 11/SC/0107).

## Abbreviations

DASH: Disabilities of the Arm, Shoulder and Hand; NHS: National Health Service; QALY: Quality Adjusted Life Year.

## Competing interests

Kim Burton was involved in the development of *The Arm Book*, to which the experimental leaflet in this trial is related: he may receive future royalties on the booklet.

## Authors’ contributions

GTJ is chief investigator of the trial, and is ultimately responsible for all aspects of trial conduct. GJM, KTP, DC, KWB, KB, PM, AM were co-applicants on the original funding application, contributed to the overall design of the trial and are jointly responsible for oversight of trial conduct. CM was a collaborator on the original funding application; PH was responsible for production of physiotherapy treatment guidelines; both also contribute to trial oversight. KB was responsible for production of the advice material – for both the active and passive advice groups. AM is the trial statistician and drafted the trial analysis plan, to which all other authors had the opportunity for critical input. KM is the trial manager responsible for day-to-day management of the trial. GTJ was responsible for drafting the manuscript. All authors read, had critical input, and approved the final manuscript.

## Pre-publication history

The pre-publication history for this paper can be accessed here:

http://www.biomedcentral.com/1471-2474/15/71/prepub

## Declaration

All the authors of the manuscript have read and agreed to its content and are accountable for all aspects of the accuracy and integrity of the manuscript in accordance with ICMJE criteria. Readily reproducible materials described in the manuscript are available to any scientist wishing to use them for non-commercial purposes – please contact the corresponding author for further information. Ethical approval for the study was obtained (see above).
